# Plant-Derived Nanocellulose with Antibacterial Activity for Wound Healing Dressing

**DOI:** 10.3390/pharmaceutics15122672

**Published:** 2023-11-25

**Authors:** Gabriela Mădălina Oprică, Denis Mihaela Panaitescu, Brînduşa Elena Lixandru, Catalina Diana Uşurelu, Augusta Raluca Gabor, Cristian-Andi Nicolae, Radu Claudiu Fierascu, Adriana Nicoleta Frone

**Affiliations:** 1 National Institute for Research and Development in Chemistry and Petrochemistry, 202 Spl. Independentei, 060021 Bucharest, Romania; madalina.oprica@icechim.ro (G.M.O.); catalina.usurelu@icechim.ro (C.D.U.); raluca.gabor@icechim.ro (A.R.G.); cristian.nicolae@icechim.ro (C.-A.N.); fierascu.radu@icechim.ro (R.C.F.); adriana.frone@icechim.ro (A.N.F.); 2 Department of Science and Engineering of Oxide Materials and Nanomaterials, National University of Science and Technology POLITEHNICA Bucharest, 1-7 Gh. PolizuStreet, 011061 Bucharest, Romania; 3 Cantacuzino National Medical-Military Institute for Research and Development, 103 Spl. Independentei, 050096 Bucharest, Romania; lixandru.brindusa@cantacuzino.ro; 4 Department of Bioresources and Polymer Science, National University of Science and Technology Politehnica Bucharest, 1-7 Gh. PolizuStreet, 011061 Bucharest, Romania

**Keywords:** cellulose nanofibers, propolis, antibacterial, sponges, wound dressing

## Abstract

The medical sector is one of the biggest consumers of single-use materials, and while the insurance of sterile media is non-negotiable, the environmental aspect is a chronic problem. Nanocellulose (NC) is one of the safest and most promising materials that can be used in medical applications due to its valuable properties like biocompatibility and biodegradability, along with its good mechanical properties and high water uptake capacity. However, NC has no bactericidal activity, which is a critical need for the effective prevention of infections in chronic diabetic wound dressing applications. Therefore, in this work, a natural product, propolis extract (PE), was used as an antibacterial agent, in different amounts, together with NC to obtain sponge-like structures (NC/PE). The scanning electron microscope (SEM) images showed well-impregnated cellulose fibers and a more compact structure with the addition of PE. According to the thermogravimetric analysis (TGA), the samples containing PE underwent thermal degradation before the unmodified NC due to the presence of volatile compounds in the extract. However, the peak degradation temperature in the first derivative thermogravimetric curves was higher for all the sponges containing PE when compared to the unmodified NC. The antibacterial efficacy of the samples was tested against *Staphylococcus aureus*, *Pseudomonas aeruginosa*, and *Escherichia coli*, as well as on two clinically resistant isolates. The samples completely inhibited the development of *Staphylococcus aureus*, and *Pseudomonas aeruginosa* was partially inhibited, while *Escherichia coli* was resistant to the PE action. Considering the physical and biological properties along with the environmental and economic benefits, the development of an NC/PE wound dressing seems promising.

## 1. Introduction

The environmental challenge we face today has led researchers and the industry to look away from petroleum-based derivatives and turn their attention to plant-based products in an effort to protect the environment and slow down climate change [1,2]. The contamination of soil and water resources with petroleum products is one of the most critical environmental problems nowadays, and the widespread use of natural products is seen as a valuable solution to preserve the environment. From the most abundant biopolymers and natural extracts, researchers try to design new products for medical and industrial applications that have some of the most popular characteristics: biodegradability, biocompatibility, and bio-origin. The most affordable and abundant biopolymer in nature, cellulose, has already established itself as a replacer for some synthetic materials [3,4]. Sourced from plants, wood, agro-industrial waste, bacteria, fungi, algae, and marine invertebrates, cellulose checks every box for the three bio characteristics [5]. Aside from its eco-friendly attribute, when cellulose is diminished to nano dimensions, a valued material, nanocellulose (NC), is obtained [1,4]. NC has a high surface area available for ulterior modifications, which is extremely useful for the diversification of its applications and endowment with new properties [6].

One of the most important applications of NC is in the biomedical sector [1,3,7]. Actually, nanomaterials found widespread applications in medicine in areas such as cancer theranostics, wound healing, drug delivery, vaccines, biosensors, and others [8]. In this sector, the wound dressing market is expected to reach USD 11.2 billion in revenue by 2025, due to the wide application, from surgical to chronic wounds [9]. Some of the best characteristics that put NC as the base for various wound dressing concepts include its capacity to absorb exudates and maintain a certain level of humidity around the wound due to its good water uptake, along with good mechanical performance [10,11]. However, a crucial property for wound dressing application is the antibacterial activity, something that NC lacks but can attain with the proper modification [12]. The antibacterial properties are essential for wound dressing materials to prevent infection spreading and speed up healing [13,14]. These problems are even more challenging for diabetic patients or those with compromised immunity because, in these cases, the infection greatly delays the healing. According to the World Health Organization, around 422 million people are affected by diabetes, especially type 2 diabetes, which is the cause of chronic wounds with an extended inflammatory phase [10,15]. The challenges regarding diabetic chronic wound treatment are numerous; therefore, the solutions should be very diverse. 

Antibacterial agents derived from plants in the form of extracts or chemically synthesized ones are very interesting since they offer endless possibilities and solutions to endow cellulose with antibacterial properties [16,17]. Propolis, one of the best glues that nature has to offer, is a secondary product of the beehive. Besides its role in fixing the hive due to its compact structure, propolis is used for its antiseptic properties in protecting against microbial infection [18]. The characteristics of propolis are influenced by the geographical position, the vegetation, or the season the bees are collecting, similar to many other natural products [18]. The complex formulation of propolis is a combination of pollen, bee wax, resins, exudates from plants, essential oils, and other organic compounds. With over 300 compounds making its structure, including flavonoids, phenolic compounds, and terpenoids, propolis is a lipophilic material [19,20]. Due to its remarkable characteristics, propolis is employed as much by humans and bees. Very important for the biological attributes of propolis are the polyphenols and flavonoid components, which give antioxidant activity, diminish lipid peroxidation, have immunomodulatory and immunostimulatory effects, as well as antibacterial action [19]. The importance of propolis in wound healing may be attributed to its bacterial and biofilm formation inhibition, which was demonstrated against staphylococci, streptococci, and some antibiotic-resistant tuberculosis strains [21]. Previous studies have demonstrated the beneficial role of propolis in nanostructured lipid carriers for stopping breast cancer evolution [22], in cellulose acetate/polyethylene oxide/hydroxypropyl methyl cellulose composite to obtain a drug-releasing resorbable suture material [23], and in dressing for wound healing based on bacterial cellulose (BC) incorporating propolis and *Cinnamomum cassia* essential oil, which, as expected, inhibited the growth of Gram-positive bacteria, while also affecting the growth of Gram-negative bacteria [16]. Propolis was also used to induce antimicrobial activity in nanocellulose-containing polymer composites [24,25]. Porous structures obtained from cellulose nanofibers (CNFs) and poly(vinyl alcohol) (PVA) were loaded with propolis extract (PE) and showed antimicrobial activity against *Escherichia coli*, *Streptococcus mutans*, and *Candida albicans* [24]. In addition, cellulose/PVA (3:1) composites containing small amounts of vitamin C and/or propolis showed a controlled release of vitamin C and an antibacterial activity against *Staphylococcus aureus* and *Escherichia coli* [25]. Based on in vitro and in vivo experiments, the composite film containing vitamin C and propolis was proposed as a new therapeutic method for healing wounds in diabetic patients [25]. 

Several studies on cellulose modified with propolis to gain antimicrobial properties were carried out starting from bacterial cellulose [26,27,28]. Composite films consisting of BC impregnated with zinc oxide (ZnO) nanoparticles and ethanolic propolis extracts (EEP) were designed for food packaging applications [26]. A synergistic effect of the ZnO/EEP antibacterial agents was observed, depending on the type of bacteria and the EEP concentration [26]. In another work, bacterial nanocellulose (BNC) hydrogels were obtained using propolis extract as an antibacterial agent along with gelling agents, preservatives, humectants, and, in some cases, methylene blue (MB) as a photosensitizer [27]. All systems having in composition PE exhibited some antimicrobial effect against *Staphylococcus aureus*, but the BNC containing MB completely eradicated the microorganisms in the presence of light due to their photodynamic inactivation effect [27]. Moreover, manually compressed BC membranes that were impregnated with different propolis fractionated extracts showed an accelerated wound healing of lesions in diabetic mice as compared to the control [28].

All studies so far report the influence of propolis or PE on the antibacterial activity of bacterial cellulose, while the straightforward plant-derived nanocellulose/PE materials have not yet been studied. However, the expensive and prolonged production cycle of BC makes it infeasible for widespread use at present [29], and the presence of residual bacterial cells encapsulated in the BC network makes their complementary sterilization process absolutely necessary for medical application [30]. Meanwhile, NC is commercially available in high quantities as CNFs or cellulose nanocrystals (CNCs), and its high surface area and easy handling make it attractive for medical applications for wound healing [31]. Moreover, NC can be obtained from different agro-industrial wastes, making it a cheap material. Therefore, in this work, plant-derived nanocellulose (NC) with antibacterial activity was obtained from nanofibrillated cellulose by impregnating it with different high proportions of PE followed by freeze-drying. The obtained sponges were characterized to emphasize the influence of PE on the mechanical, thermal, and morphological features of the NC sponges in addition to their antibacterial activity. 

## 2. Materials and Methods

### 2.1. Materials

Microcrystalline cellulose (MCC) with 20 µm mean particle size was purchased from Sigma Aldrich (St. Louis, MO, USA) and used as a base for obtaining a nanocellulose via the microfluidization of a 1.5 wt% MCC aqueous suspension in fifteen successive cycles using a LM20 Microfluidizer (Microfluidics International Corporation, Westwood, MA, USA). Propolis extract in ethanol (PE), containing 20% propolis, was purchased from ApiLand (Baia Mare, Romania) and subsequently filtrated through a filter paper to remove any remaining solid part. 

### 2.2. Preparation of Nanocellulose Sponges

**Figure 1:**
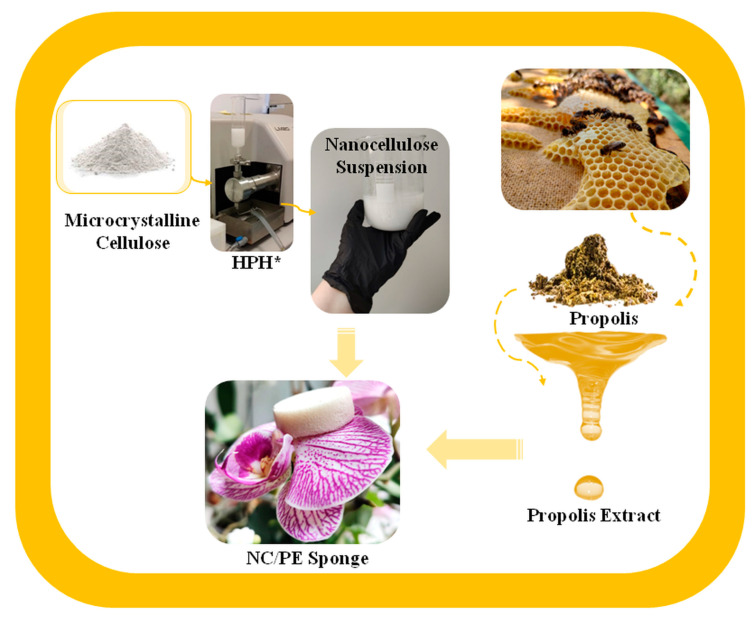
Schematic representation of the phases involved in obtaining the NC/PE sponges, where HPH* represents high-pressure homogenization.

The sponges were obtained using a nanocellulose suspension in water (1.5 wt%) and different amounts of PE (Table 1). The mixtures were kept on a magnetic stirrer set at approximately 600 rpm, at room temperature, for 10 h for good homogenization. Then, the samples were stored in the freezer at −20 °C for 24 h and freeze-dried at −85 °C under vacuum (72 h) in a FreeZone 2.5 L Benchtop Freeze Dry System (Laboconco, Kansas City, MO, USA). The schematic representation of the phases involved in the production of the NC/PE sponges is shown in Figure 1. 

### 2.3. Characterization

#### 2.3.1. Fourier-Transform Infrared Spectroscopy (FTIR) Spectroscopy

The structural characteristics of pristine nanocellulose, dried propolis extract, and NC/PE sponges were analyzed via FTIR, Attenuated Total Reflectance (ATR) mode, using a Bruker Tensor 37 spectrophotometer (Waltham, MA, USA). The recordings were collected in the 4000–500 cm^−1^ spectral range, with 16 scans and a 4 cm^−1^ resolution.

#### 2.3.2. Thermogravimetric Analysis (TGA)

The thermal analysis was carried out for all the PE-containing sponges, the propolis extract, and the unaltered nanocellulose. For this purpose, a TGA Q5000 from TA Instruments (New Castle, DE, USA) was used in the 25–750 °C temperature range, with a 10 °C/min heating rate under nitrogen flow (40 mL/min).

#### 2.3.3. Scanning Electron Microscopy (SEM)

A Hitachi TM4000 plus scanning electron microscope (Hitachi, Tokyo, Japan) was used to investigate the morphology of the NC/PE sponges and the influence of the PE on the NC structure. For this purpose, the NC/PE sponges, as well as the pristine NC, were sputter coated in a Q150R Plus (Quorum Technologies, SXE, Lewes, UK) with a gold layer of 5 nm for higher-quality images. 

#### 2.3.4. Dynamic Mechanical Analysis (DMA)

A DMA Q800 dynamic mechanical analyzer from TA Instruments (New Castle, DE, USA) was used to measure the mechanical properties of the sponges. Cylindrical-shaped samples, with the approximate thickness × diameter of 10 mm × 35 mm, were tested to a maximum force of 18 N, with a 0.5 N/min advance. 

#### 2.3.5. Antibacterial Test

Fragments of approximately the same size were cut from the test samples, NC/PE 4/2, NC/PE 4/3, and NC/PE 4/4, while NC/PE 4/0 was used as a negative control, employing a sterile scalpel to avoid microbial contamination during handling. The fragments were placed in Petri dishes and sterilized under UV light for 15 min. In vitro antimicrobial activity of the sponges was screened using the disc diffusion method [32,33] against three reference bacterial strains, *Staphylococcus aureus* ATCC29213 (*S. aureus*), *Escherichia coli* ATCC25922 (*E. coli*), and *Pseudomonas aeruginosa* ATCC27853 (*P. aeruginosa*), and two clinical resistant isolates, a Methicillin Resistant *Staphylococcus aureus* (MRSA) isolated from furuncle and an Extended Spectrum Beta-Lactamase (ESBL) producing *Escherichia coli* clinical isolates harboring CTX-M-1 encoding gene. The isolates stored in brain heart infusion broth with 20% glycerol at −70 °C in a deep freezer were subcultured on Columbia Agar (OXOID) supplemented with 7% defibrinated sheep blood at 37 °C for 24 h. The isolates were provided by the culture collection of Nosocomial and Antimicrobial Resistant Infections Laboratory from “Cantacuzino” National Medico-Military Institute for Research and Development (Bucharest, Romania). Bacterial inocula were prepared in sterile saline physiological water (0.85% NaCl) (*w*/*v*) and calibrated to the 0.5 McFarland standard density corresponding to the 1.5 × 10^8^ CFU (colony forming unit)/mL.

The disc diffusion method was applied against the five bacterial strains using duplicate samples. Mueller–Hinton agar plates were inoculated by applying the lawn culture technique and using a sterile cotton swab dipped into the inocula, while the excess medium was removed. The plates containing the bacteria were incubated at 37 °C for 24 h and at 4–8 °C for 5 days. After incubation, the growth inhibition zones were measured around the cellulose samples, from the edge of the sample to the complete inhibition of bacterial growth visible to the naked eye, using a caliper, and the results were expressed in mm. 

Nanocellulose and 6 mm discs of Whatman filter paper (blank discs) were impregnated with known concentrations of antibiotics and used as positive controls. They were tested against *S. aureus* ATCC29213 and MRSA. The positive control plates were obtained in the same conditions as those for NC/PE samples. The antibiotics loaded on the NC and the Whatman discs were as follows: cefoxitin (FOX, 30 µg), azithromycin (AZT, 15 µg), teicoplanin (TEC, 30 µg), minocycline (MH, 30 µg), and amikacin (AK, 30 µg). 

## 3. Results and Discussion

### 3.1. Fourier-Transform Infrared Spectroscopy (FTIR) Spectroscopy

**Figure 2:**
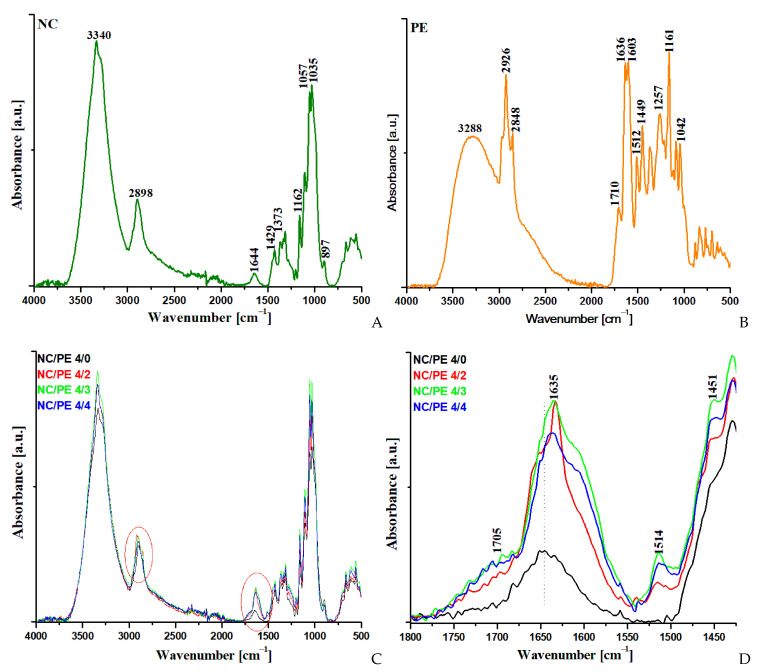
FTIR spectra for pristine NC (**A**), dried propolis extract (**B**), NC/PE sponges (**C**), and detail in the region 1800–1425 cm^−1^ (**D**); the red circled areas in Figure 2C show the zones where the presence of propolis’ vibrations in the FTIR spectra for NC/PE sponges is most evident.

The FTIR spectra of the NC/PE sponges, NC reference (NC/PE 4/0), and propolis reference are shown in Figure 2A–C. The propolis reference was obtained from the PE after drying at room temperature for two weeks. The well-known FTIR spectrum of NC from Figure 2A shows the most imposing absorption band between 3650 and 2995 cm^−1^, which is attributed to the stretching vibrations of the hydrogen-bonded –OH groups on the cellulosic surface, an absorption band registered between 2990 and 2800 cm^−1^, which is assigned to the stretching vibrations of the –C–H bonds, and a small absorption at around 1644 cm^−1^, which is characteristic to the bound water in NC [34,35,36]; other absorption peaks were observed at 1429 cm^−1^, assigned to –CH wagging, and 1373 cm^−1^, assigned to –CH bending [15,35]. The C–O–C asymmetric bridge stretching was observed at 1162 cm^−1^, the C–O (secondary alcohol) stretching vibrations at 1057 cm^−1^, and the C–O (primary alcohol) stretching vibrations at 1035 cm^−1^ [34,37]. The β-glycosidic link between the glucose units is shown by a peak at 897 cm^−1^ [15,36,38].

Several differences were observed in the FTIR spectra of the NC/PE sponges as compared to that of neat NC (NC/PE 4/0) (Figure 2C): a distinctive peak at around 1514 cm^−1^, which is attributed to the double carbon bonds of an aromatic ring [39,40], a shoulder at 1451 cm^−1^, which is characteristic to C-H bending and aromatic stretching, a new sharp peak at 1635 cm^−1^ that can be assigned to C=O stretching and C=C stretching as well as to N–H asymmetric bending [41], and a small broad shoulder at around 1705 cm^−1^, which is assigned to C=O stretching [18,41]. All these differences are due to the presence of propolis in the NC/PE sponges, as it can be easily demonstrated by analyzing the FTIR spectrum of propolis (Figure 2B). Thus, the main peaks of propolis are located between 3700 and 3000 cm^−1^, with a maximum at 3288 cm^−1^, which can be attributed to the stretching vibrations of the hydroxyl group from alcohols and phenols [39], at 2926 and 2848 cm^−1^, which correspond to the C–H symmetric and asymmetric stretching vibrations in methyl, methylene, and methine groups and is due to the presence of carbohydrates [18,42], at 1710 cm^−1^ due to the C=O stretching in fatty acids and flavonoids [18,41], at 1636 cm^−1^ due to the C=O and C=C stretching and N–H bending in flavonoids and amino acids [39,41], at 1603 cm^−1^ and 1512 cm^−1^(C=C stretching in the aromatic ring [39,41]), at 1449 cm^−1^ (C–H bending in flavonoids and aromatic ring [41]) and at 1161 and 1042 cm^−1^ due to C-O stretching vibrations in lipids and alcohol groups [41]. Although the presence of the peaks at around 3300 and 2900 cm^−1^ in the sponges may be overlapped by the characteristic peaks of cellulose in these regions, the peaks at 1710, 1636, 1512, and 1449 cm^−1^, which are characteristic of flavonoids, lipids, and amino acids in propolis, can be observed in the spectra of NC/PE sponges at close wavelengths (Figure 2C,D). 

### 3.2. Scanning Electron Microscopy (SEM)

**Figure 3:**
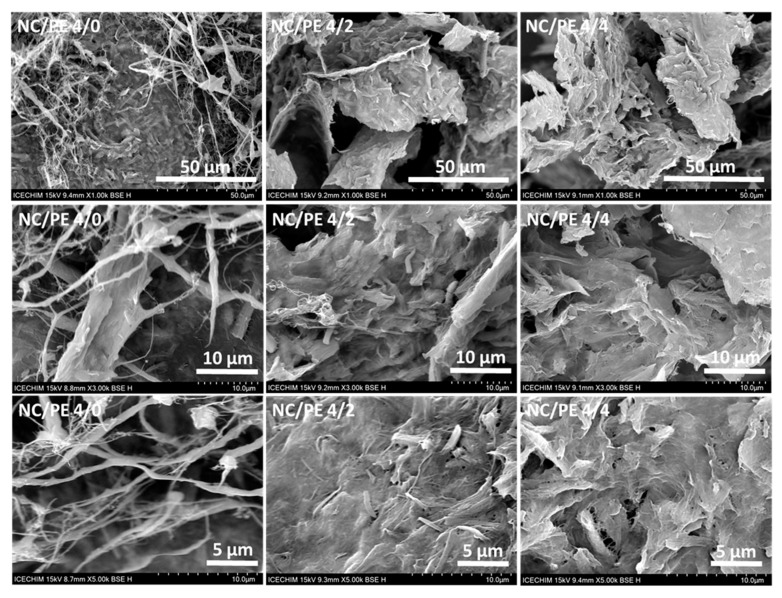
SEM images for NC/PE 4/0, NC/PE 4/2, and NC/PE 4/4 at different magnifications, ×1000, ×3000, and ×5000.

The SEM images of the NC/PE 4/2 and NC/PE 4/4 sponges were compared to those of unmodified NC/PE 4/0 in Figure 3 to emphasize the changes in the surface morphology of the sponges following the addition of the propolis.

The SEM images of the unmodified NC (NC/PE 4/0) reveal that the NC sponges consist of long individual nanofibers with a thickness < 100 nm and strands with micrometric thickness formed by tens of nanofibers along with a compact network of nanofibers with an entangled structure and nanometer-sized pores. This morphology is the result of the freeze-drying process, which favors the aggregation of nanofibers in the interstitial regions between the ice crystals [43]. The addition of propolis led to important morphological changes in the sponges, as illustrated by the SEM images in Figure 3. Individual fibers and strands rarely appear in the SEM images of the NC/PE 4/2 and NC/PE 4/4 sponges, and a compact network consisting of entangled nanofibers has the tendency to occupy the entire surface (Figure 3). Although the SEM images of all the sponges under lower and medium magnification show the presence of large pores and interconnected channels of more than 50 µm in size, regardless of the presence or concentration of PE, the channel walls appear more compact and the pores rarer in the sponges containing PE. The capacity of the propolis extract to inflict a more compact structure and a decrease in the pore size was also observed in hydrogels based on cellulose nanofibers and poly (vinyl alcohol) [24]. Moreover, looking at higher magnification images, the NC/PE 4/2 and NC/PE 4/4 sponges have a neater appearance than the NC/PE 4/0, with the cellulose nanofibers intertwined closely with each other in a random manner. Similarly, the electrospun cellulose acetate (CA)/polycaprolactone (PCL) nanofibers containing propolis showed a smoother appearance when impregnated with propolis extract due to its adhesive properties [44]. No individual propolis particles are visible in these images, suggesting good wetting of the cellulose fibers with the PE and its deep penetration into the fiber network. 

### 3.3. Thermogravimetric Analysis (TGA)

**Figure 4:**
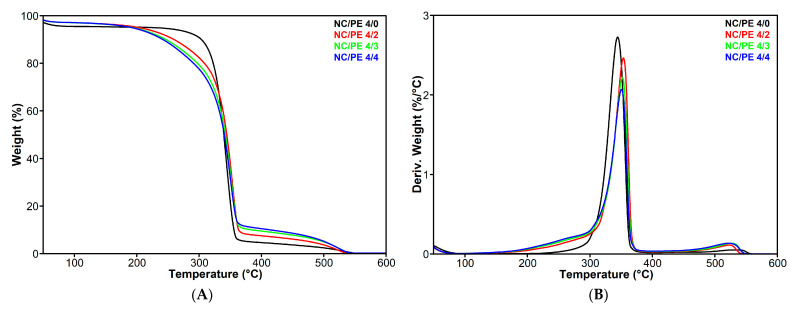
Thermogravimetric analysis results, TG (**A**) and DTG (**B**), for the NC/PE sponges.

The thermal stability of propolis is low, as it begins to release water and volatile compounds just above room temperature [45,46,47]. Therefore, propolis addition in NC sponges could influence their thermal stability, which is an important characteristic for biomedical and food applications. The thermal behavior of the NC/PE sponges was monitored using TGA, with the curves corresponding to the TG (the mass loss against temperature) and the first derivative of the TG curve as a function of temperature (DTG) presented in Figure 4. The characteristics that highlight the thermal stability of the sponges, i.e., the temperature registered at 5% weight loss (T_5%_), the onset degradation temperature (T_on_), the temperature at the maximum degradation rate (T_max_), and the residue registered at 600 °C (R_600°C_), are listed in Table 2.

The first thermal event registered up to 100 °C for all the sponges represents the evaporation of the water absorbed in the spongious structures [48]. The weight loss at 100 °C was 4.5% for the unmodified NC (NC/PE 4/0) and only 3% for the sponges impregnated with PE. The main thermal event for the unmodified NC was observed between 290 and 360 °C with a decomposition peak at 345 °C in its DTG thermogram (Figure 4). This degradation step corresponds to the breaking of glycosidic bonds and depolymerization reactions along with the decomposition of the cyclic structures and the release of volatiles, especially methane and carbon oxides, which result in the formation of char residue [49,50,51]. However, the sponges containing different amounts of propolis show an additional shoulder in the DTG spectra between 200 and 300 °C. This new thermal event is attributed to the degradation of free amino acids and polyphenols present in propolis [45]. The analysis of the T_5%_ and T_on_ values (Table 2) could give information on the intensity of this thermal event. It may be remarked that the order in which the NC/PE sponges enter the degradation process is opposite to the increase in propolis concentration, T_5%,_ and T_on_ increasing in the order NC/PE 4/4 <NC/PE 4/3 <NC/PE 4/2. A similar behavior was reported for PVA/propolis nanofibers, which were electrospun on a commercial Ni–Ti stent [46]. The decomposition temperature of PVA/propolis composites dropped sharply with increasing the propolis content from 0 to 4% [46]. In addition, bacterial nanocellulose hydrogels containing different amounts of propolis and methylene blue as a photosensitizer, which were developed for wound healing, showed a decrease in thermal stability with the increase in propolis content [27].

Remarkably, although the NC sponges impregnated with propolis showed a lower thermal stability at temperatures below 300 °C, the temperature at maximum degradation rate (T_max_) increased with about 6 °C from NC/PE 4/0 to NC/PE 4/4. It may be observed in Figure 4 that the main degradation process of cellulose is faster for the unmodified NC than for the sponges impregnated with propolis, which suggests that propolis delays the degradation of cellulose. Such a thermal behavior of cellulose-based materials was hardly found in the literature. A starch/carboxymethyl chitosan film containing different amounts of dried PE showed only for the maximum concentration of PE (10%) an increase in the T_max_ by 19 °C, although the degradation of propolis induced a faster degradation of the compounds before 200 °C [52]. This behavior could be determined using the barrier created from the propolis coating at the surface of cellulose nanofibers, as revealed in the SEM images of the NC/PE sponges. However, chemical reactions involving the OH/COOH groups from the cellulose surface and the amino acids or other components from the PE can be also considered as possible at the high temperature attained during the measurement [45]. 

Another decomposition step was observed in the DTG curves of all samples between 450 and 550 °C (Figure 4). This is the carbonization stage, where the pyrolysis intermediates suffer further decomposition leaving behind a residue consisting of coke and graphite [50,53]. The residue at 600 °C, registered for the NC/PE samples, increases with the increase of propolis amount, but the increase was not proportional to the PE concentration being more abrupt between the sample without (NC/PE 4/0) and the one with the minimum amount of propolis (NC/PE 4/2) (Table 2). This was probably determined using the high solid residue of the propolis that exceeds 20% [45]. 

### 3.4. Dynamic Mechanical Analysis (DMA)

**Figure 5:**
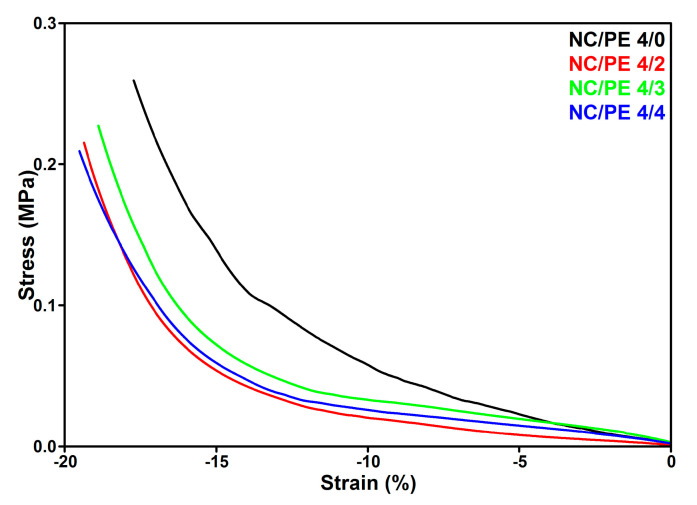
Compression stress–strain curves for the NC/PE sponges.

The influence of PE on the mechanical properties of the NC/PE sponges was determined via DMA measurements in compression mode. The data collected from these measurements, i.e., stress vs. strain values, are shown in Figure 5.

At very low strains, the difference between the mechanical behavior of the sponges is small, and the NC/PE 4/3 shows the highest mechanical strength. At higher strains, the influence of the PE on the behavior of the sponges during compression is more apparent. Lower strength values at the same strain were observed in Figure 5 for the sponges containing propolis compared to the unmodified NC sponge. The concentration of PE in the NC/PE sponges does not have much influence on their mechanical properties. At the 10% strain, the compression stress decreased from 57 kPa for unmodified NC (NC/PE 4/0) to 21 kPa for NC/PE 4/2, 34 kPa for NC/PE 4/3, and 27 kPa for NC/PE 4/4. However, the values obtained for the NC/PE sponges containing propolis were higher than those reported for cellulose sponges obtained from a cotton/microcrystalline cellulose mixture modified with natural antibacterial extracts, which presented compression strength values lower than 10 kPa at 10% strain [54] and similar to those reported for bacterial cellulose scaffolds modified with gelatin via procyanidins crosslinking technique and hydroxyapatite-coating, which presented compression strength values around 20 kPa [55]. Therefore, the mechanical properties of the NC/PE sponges could be considered adequate for medical application. However, the decrease in the compression strength is an effect of propolis that covers the cellulose fibers and bonds them forming “walls”, which no longer offer the same resistance to the action of the compression force due to the lack of contact between them on large areas. Thus, the mechanical behavior of the sponges containing PE is proof of good coverage of the cellulose fibers, which was also demonstrated in the SEM analysis that revealed important morphological changes in these sponges. 

### 3.5. Antibacterial Efficacy Assessment

The in vitro antibacterial activity of NC/PE 4/2, NC/PE 4/3, and NC/PE 4/4 sponges was assessed along with a negative control represented by untreated nanocellulose (NC/PE 4/0) against *Staphylococcus aureus* ATCC 29213, *Escherichia coli* ATCC 25922, and *Pseudomonas aeruginosa* ATCC 27853. Two clinical resistant isolates, an MRSA and an ESBL-producing *Escherichia coli*, were also used to evaluate the antibacterial activity of the sponges. Table 3 shows the sizes of the growth inhibition zones for each bacterial strain—propolis containing sample pair having antibacterial activity. Untreated nanocellulose sponge (NC/PE 4/0), serving as a negative control, showed no antibacterial activity, as previously demonstrated for bacterial cellulose [16,47] and plant-derived cellulose [56]. The growth inhibition zone results displayed in Table 3 represent the average distance in mm measured from the edge of the sample to the edge of the inhibition zone along with the standard deviation. 

**Figure 6:**
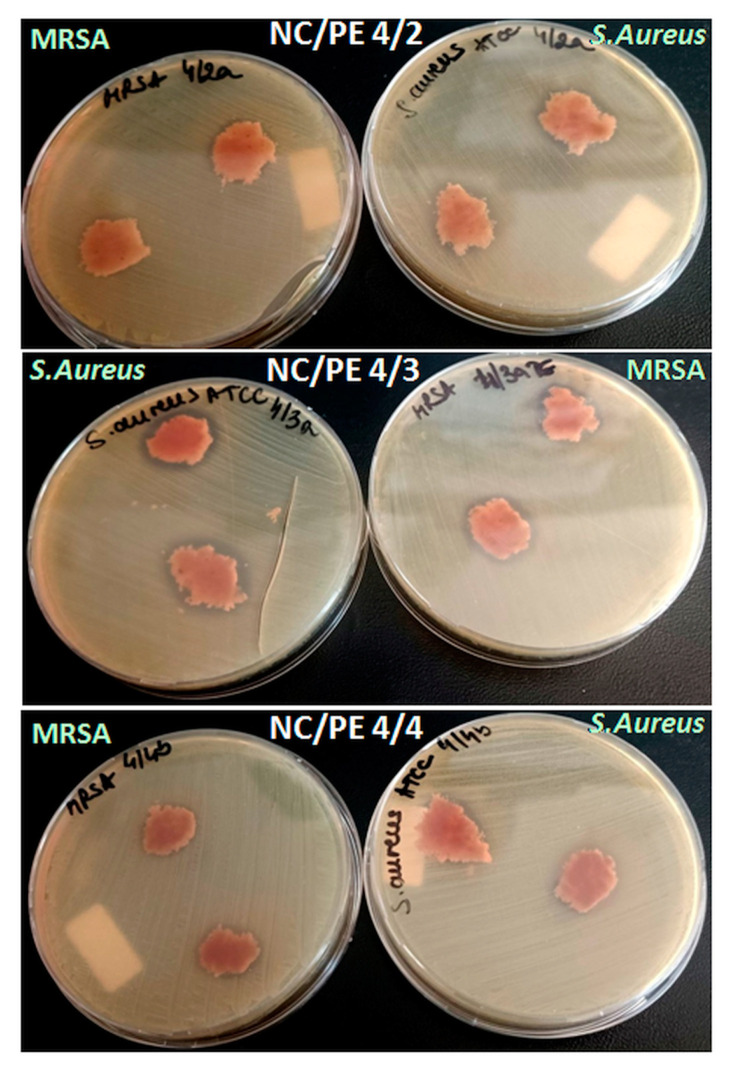
Images showing the growth inhibition zones for NC/PE sponges against *S. aureus* and MRSA.

All tested NC/PE sponges were found to present a complete growth inhibition against *S. aureus* and MRSA strains (Figure 6). The antimicrobial activity of the sponges was, however, not proportional to the PE amount, with NC/PE 4/3 showing the highest inhibitory activity. Similar results were reported for rice starch/carboxymethyl chitosan (RS/CMCh) films containing PE, where the inhibition zone measured from the edge of the sample (~1.1 mm) was close for the films containing 5 and 10% PE [52]. The antibacterial activity of the NC/PE sponges is due to the active compounds in propolis, such as flavonoids, aromatic compounds, and terpenoids, which have the ability to destroy the cell structure [46]. Previous studies have shown that quercetin, apigenin, caffeic acid, and coumaric acid, which are all components of propolis, are responsible for decreasing bacterial mobility and adenosine triphosphate production, for the inhibition of bacterial cell division and the increase in the cell membrane permeability [57,58]. Moreover, propolis is characterized by a high total phenol content of about 237.0 mg gallic acid equivalents (GAE)/g of the sample when it was collected from Chiang Mai Province (Thailand) [52] or 333.8 GAE/g for propolis collected from Ineu, Arad (Romania) [59], which may explain its inhibition effect against *S. aureus* and MRSA strains. 

**Figure 7:**
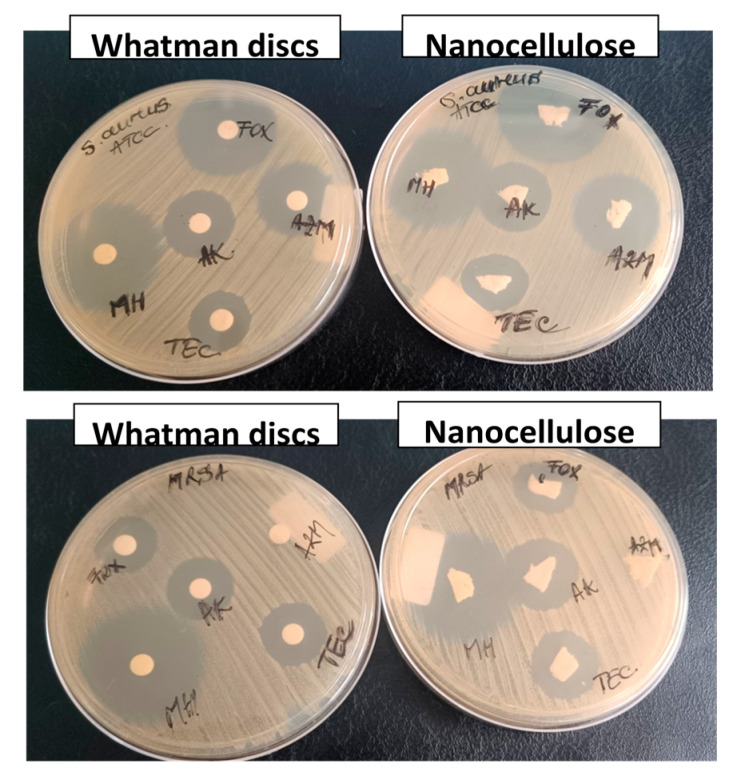
Images showing the growth inhibition zones for nanocellulose and Whatman discs impregnated with high concentrations of cefoxitin (FOX), azithromycin (AZT), teicoplanin (TEC), minocycline (MH) and amikacin (AK) against *S. aureus* and MRSA.

For comparison, the antibacterial activity of the antibiotics against *S. aureus* ATCC 29213 and the MRSA clinical strain is shown in Figure 7. Similar inhibition zones were observed for the same antibiotic in the case of nanocellulose and Whatman discs but a different activity against *S. Aureus* and MRSA for the different antibiotics, as expected. 

**Figure 8:**
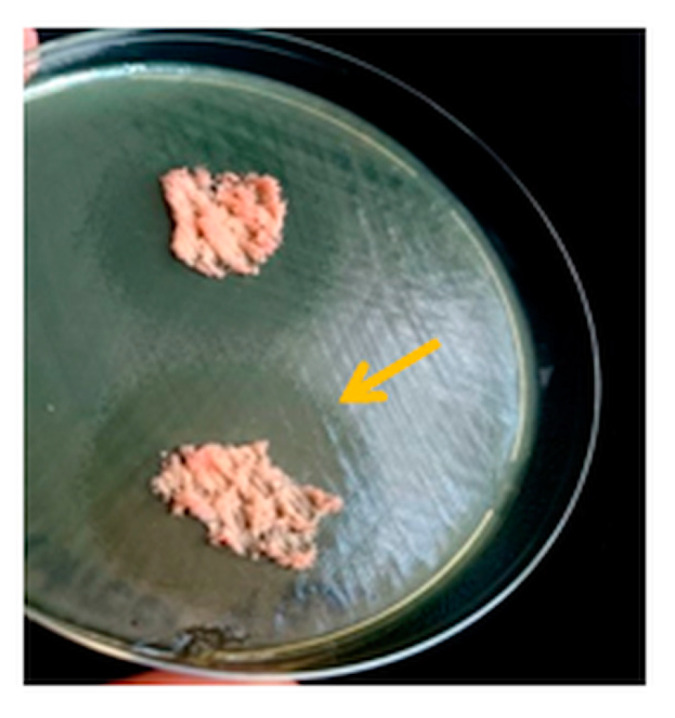
Image showing the antibacterial activity of the NC/PE 4/4 sponge against *P. aeruginosa*.

A remarkable inhibition zone of 32–35 mm was obtained against *P. aeruginosa* ATCC 27,853 for all tested NC/PE sponges (Table 3, Figure 8). However, the growth inhibition was incomplete in these cases; the observed *ghost zones* consist of large clear zones with poorer culture growth between the NC/PE sample and the edge of the inhibition zone. This *ghost zone* is suggestive of a decrease in the number of tested bacteria. Incomplete inhibition was previously reported in the case of acrylic bone cement loaded with two different antibiotics for the treatment of *Pseudomonas aeruginosa* in joint arthroplasty [60] or for the *Lepidospermaviscidum* plant extract against vancomycin-resistant enterococci [61]. 

The results showed that the *E. coli* clinical isolate (ESBL-producing *Escherichia coli*) and *E. coli* ATCC 25922 quality control strain were resistant to the antimicrobial activity of the propolis extract, revealed by the absence of inhibition zones. A similar result was reported for the RS/CMCh films where no inhibition zone was detected for *E. coli,* regardless of the PE concentration, while the films containing 5 and 10% PE inhibited *S. aureus* growth [52]. Moreover, a limited antibacterial activity of propolis has been previously reported against *E. coli* and a good one against *S. aureus* [62]. In general, a more intense antibacterial activity against Gram-positive bacteria than Gram-negative bacteria has been previously reported for plant extracts [63]. This was explained by the fact that, unlike Gram-positive bacteria, *E. coli* bacteria have a thicker cell wall containing an additional outer lipid membrane that is hardly penetrated by the antibacterial agent [52,64]. A possible mechanism of the lower activity of propolis on Gram-negative bacteria was considered to be the synthesis by Gram-negative bacteria in their outer membrane of a wide variety of hydrolytic enzymes that interfere with the active compounds of propolis, leading to the emergence of resistance [65]. Other studies suggested that the barrier function of the Gram-negative outer membrane is species dependent, possibly reflecting the porin or lipopolysaccharide differences in the outer membrane [66]. Moreover, it was assumed that the relatively higher resistance of *E. coli* to propolis extracts may be due to the lack of an effective target site in the metabolic pathways or cellular structural components required for the destruction action of propolis [67].

However, *P. aeruginosa* belongs to Gram-negative bacteria similar to *E. coli* and is characterized by a 100 times lower permeability outer membrane than *E. coli* [68], while NC/PE sponges showed partial growth inhibition against *P. aeruginosa* and no inhibition against *E. coli*. This result is hard to explain based on the current knowledge regarding the interactions between the active components of propolis and different Gram-negative bacteria. However, considering the four main components of the Romanian propolis, i.e.,kaempferol, quercetin, resveratrol, and rosmarinic acid [59], a different effect of each component on the inhibition of *P. aeruginosa* and *E. coli* was observed. Thus, a plant extract of *Artemisia cina* (KJT) (100 mg/L ethyl acetate) characterized by a high content of kaempferol showed a slightly higher inhibition zone against *P. aeruginosa* than against *E. coli*, while a similar amount of *Artemisia cina*(J) extract with high content ofquercetin had an opposite effect [69]. Similarly, it has been reported that resveratrol inhibited the growth of *P. aeruginosa*, but it was metabolized by *E. coli* [70]. Therefore, besides the biological differences between strains, the interactions between the active components in propolis, which greatly differ depending on the geographical area and climate, and microorganisms are very important, and their further study is particularly important for the design of new antibacterial dressings and drugs.

In summary, the antibacterial assay showed that the NC/PE 4/3 sponge presents the best antibacterial properties together with the best mechanical behavior and good thermal stability. This formulation may be considered a promising material for wound healing applications. 

## 4. Conclusions

The aim of this study was to endow nanocellulose with antibacterial activity, which is a critical need for the effective prevention of infections in chronic diabetic wound dressing applications. Propolis, a natural derivate with known bioactive roles, was used in this work to provide antibacterial activity to plant-extracted nanocellulose sponges. The antibacterial nanocellulose sponges, which are intended for wound dressing applications, were obtained using a simple impregnation method. The SEM results showed a good impregnation of the propolis extract on the surface of the cellulose nanofibers and a densely packed structure of the sponges after the addition of propolis compared with the unmodified NC sponge, where loose fibers were observed. The thermal behavior of NC was only slightly influenced by the addition of PE, with the NC/PE 4/4, NC/PE 4/3, and NC/PE 4/2 sponges undergoing thermal degradation before NC/PE 4/0 due to the higher accumulation of volatile compounds from propolis. The antibacterial tests showed the ability of PE to inhibit the growth of *S. aureus* and MRSA, regardless of the propolis amount in the sponges. The development of a PE-based nanocellulose wound dressing showing antibacterial activity seems very promising, considering the preservation of the structure and special properties characteristic to NC while acquiring antibacterial activity. 

## Figures and Tables

**Table 1:** The composition of the nanocellulose sponges considering the mass of the NC suspension (m_NCsuspension_), the mass of the propolis extract (m_PE_) together with the mass of neat cellulose (m_NC_), and that of neat propolis (m_propolis_).

Sample	m_NC suspension_ (g)	m_NC_ (g)	m_PE_ (g)	m_propolis_(g)
NC/PE 4/0	50	0.750	-	-
NC/PE 4/2	50	0.750	1.875	0.375
NC/PE 4/3	50	0.750	2.813	0.563
NC/PE 4/4	50	0.750	3.750	0.750

**Table 2:** Characteristic data following the TGA analysis for the NC/PE sponges.

Sample	T_5%_ (°C)	T_on_ (°C)	T_max_ (°C)	R_600°C_ (%)
NC/PE 4/0	225.0	323.1	344.6	0.068
NC/PE 4/2	203.9	326.2	353.9	0.147
NC/PE 4/3	193.3	322.1	352.0	0.150
NC/PE 4/4	191.5	319.1	350.6	0.159

**Table 3:** Antibacterial activity of NC/PE samples determined using inhibition zone method.

Samples	Strain	Growth Inhibition Zones [mm]
NC/PE 4/0	*Staphylococcus aureus* ATCC 29213	no inhibition zone
NC/PE 4/2	*Staphylococcus aureus* ATCC 29213	2.5 ± 0.3
NC/PE 4/3	*Staphylococcus aureus* ATCC 29213	3.0 ± 0.1
NC/PE 4/4	*Staphylococcus aureus* ATCC 29213	2.1 ± 0.3
NC/PE 4/0	MRSA clinical isolate	no inhibition zone
NC/PE 4/2	MRSA clinical isolate	2.0 ± 0.4
NC/PE 4/3	MRSA clinical isolate	3.0 ± 0.3
NC/PE 4/4	MRSA clinical isolate	1.5 ± 0.3
NC/PE 4/0	*Pseudomonas aeruginosa* ATCC 27853	no inhibition zone
NC/PE 4/2	*Pseudomonas aeruginosa* ATCC 27853	32.0 ± 0.7 *
NC/PE 4/3	*Pseudomonas aeruginosa* ATCC 27853	35.0 ± 0.0 *
NC/PE 4/4	*Pseudomonas aeruginosa* ATCC 27853	34.5 ± 0.7 *

* incomplete growth inhibition.

## Data Availability

All the data were presented in this study.
